# Peripheral vein infusion of autologous mesenchymal stem cells in Egyptian HCV-positive patients with end-stage liver disease

**DOI:** 10.1186/scrt459

**Published:** 2014-05-28

**Authors:** Hosny Salama, Abdel-Rahman N Zekri, Eman Medhat, Shereen A Al Alim, Ola S Ahmed, Abeer A Bahnassy, Mai M Lotfy, Rasha Ahmed, Sherief Musa

**Affiliations:** 1 Hepatology and Tropical Medicine Department, El-Kasr Al-Aini School of Medicine, Cairo University, Cairo, Egypt; 2 Virology and Immunology Unit, Cancer Biology Department, National Cancer Institute, Cairo University, Cairo, Egypt; 3 Pathology Department, National Cancer Institute, Cairo University, Cairo, Egypt

## Abstract

**Introduction:**

We have assessed the utility of autologous mesenchymal stem cell (MSC) peripheral vein infusion as a possible therapeutic modality for patients with end-stage liver diseases.

**Methods:**

Forty patients with post-hepatitis C virus (HCV) end-stage liver disease were randomized into two groups: Group 1 (GI): 20 patients who received granulocyte colony-stimulating factor (G-CSF) for 5 days followed by autologous MSCs peripheral-vein infusion and group 2 (GII): 20 patients who received regular liver-supportive treatment only (control group).

**Results:**

In MSC-infused patients (GI), 54% showed near normalization of liver enzymes and improvement in liver synthetic function. Significant changes were reported in albumin (*P* = 0.000), bilirubin (*P* = 0.002), increased international normalized ratio (INR) (*P* = 0.017), prothrombin concentration (*P* = 0.029) and alanine transaminase (ALT) levels (*P* = 0.029), with stabilization of clinical and biochemical status in 13% of cases. None of the patients in GII showed any significant improvement. Hepatic fibrosis was assessed in GI by detection of procollagen IIIC peptide level (PIIICP) and procollagen III N peptide level (PIIINP). The pretreatment values of s-PIIICP and s-PIIINP were 9.4 ± 4.2 and 440 ± 189, respectively, with a decrease to 8.1 ± 2.6 and 388 ± 102, respectively, 3 months after MSC therapy. However, the difference was statistically nonsignificant (*P* = 0.7). A significant correlation coefficient was reported after 3 months between the s-PIIINP and prothrombin concentration (*P* = -0.5) and between s-PIIICP and ascites (*P* = 0.550).

**Conclusions:**

First, autologous MSC infusion into a peripheral vein is as effective as the previously reported intrahepatic infusion. Second, MSCs have a supportive role in the treatment of end-stage liver disease, with satisfactory tolerability and beneficial effects on liver synthetic functions and hepatic fibrosis. Third, IV infusion of MSCs after G-CSF mobilization improves s-albumin within the first 2 weeks and prothrombin concentration and alanine Taransaminase after 1 month. According to the data from this current study and those previously reported by our group, we recommend further studies on patients’ infusion with pure CD133 and CD34 followed by IV infusion of *in vitro*-differentiated MSCs within 1 week and another infusion after 3 months.

**Trial registration:**

ClinicalTrials.gov NCT01729221. Registered 17 November 2012.

## Introduction

Cirrhosis is the end stage of chronic liver injury caused by viral hepatitis or alcohol intake. In Egypt, which has a very high incidence of hepatitis C virus (HCV) infection, most malignant neoplasms of the liver arise on top of chronically damaged livers, typically a cirrhotic liver [1].

Although liver transplantation is considered the most effective treatment for those patients, it is critically limited by the shortage of available donors. Until now, effective treatments capable of reversing cirrhosis have not been developed. However, in the last few years, increasing evidence suggests that adult cells have greater differentiation plasticity than previously thought, with bone marrow (BM) turning into skeletal muscles [2] brain [3], or liver [4].

Several previous studies demonstrate that hematopoietic BM stem cells transplantation improves the residual liver function in cirrhosis patients. These results were considered promising and encouraging [5,6]. The bone marrow is a reservoir of various stem cells, including hematopoietic stem cells (HSCs) and mesenchymal stem cells (MSCs). Because the bone marrow-derived MSCs were found to have differentiative plasticity, great interest has occurred in their potential therapeutic application [7]. The MSCs are capable of mesodermal and neuroectodermal differentiation, they have the potential of endodermal differentiation, and their differentiation into functional hepatocyte-like cells has also been demonstrated. Nevertheless, studies on MSCs as a potential treatment for chronic liver diseases are not as advanced as those in other fields [8]. We previously showed that a CD34+ and CD133+ stem cells infusion can be used as supportive treatment for end-stage liver disease in the Egyptian population with satisfactory tolerability [9,10]. In the current study, we sought to assess the possibility of using MSCs followed by G-CSF mobilization intravenous infusion as a therapeutic modality in patients with end-stage liver diseases in comparison to our previous studies using CD133 and CD34 intrahepatic infusion.

## Patients and methods

### Studied groups

This prospective study included 40 patients with post-HCV end-stage liver disease who were eligible for treatment intervention. Patients were recruited from the Hepatology outpatient clinics at Kasr El Aini hospitals and the National Cancer Institute, Cairo University, Cairo, Egypt, during the period from June 2010 to October 2011. The enrolled patients were randomized into one of two groups; Group 1 (**GI**, the tested group) included 20 patients who received granulocyte colony-stimulating factor (G-CSF) (Neupogen;/Roche) for 5 days, followed by autologous mesenchymal stem cell (MSC) infusion, and Group 2 (**GII**, the control group) included 20 patients who received regular liver treatment. All patients had abnormal s-albumin, bilirubin, and INR values, Childs-Pugh scores B and C, a World Health Organization (WHO) performance status of 3 or higher, were unable to receive a liver transplantation because of organ shortage and the high cost of liver transplantation in Egypt, and all have the ability to give an informed consent to the procedure. All patients (MSCs transplantation and the control group) were followed up for at least 26 weeks or until they died.

### Inclusion criteria

Patients included in the study fulfilled the following criteria: male or female, aged from 20 to 60 years with evidence of chronic liver insufficiency (decreased s-albumin and/or increased bilirubin and/or increased INR), unlikely to receive a liver transplant, have a World Health Organization (WHO) performance score of less than 2 and are able to give a written consent. None of the patients has received interferon or other therapy within 6 months before cell transplantation or during the follow-up period. All patients were negative for hepatic fibrosis and for portal-tract thickening that is characteristic of schistosomiasis.

### Exclusion criteria

Patients were excluded from the study if younger than 20 or more than 60 years old; pregnant or lactating; had recent and/or recurrent upper gastrointestinal bleeding or spontaneous bacterial peritonitis (SBP) within 1 month before the procedure or HCC, an evidence of human immunodeficiency virus or other life-threatening infection; unable to give a written consent; have a history of hypersensitivity to G-CSF, or included in any other clinical trial within the previous month.

Patients in both groups were comparable for baseline characteristics including age and sex, etiology of liver disease, and MELD score. Clinical evaluation was done for all patients, including a detailed medical history and complete clinical examination with special emphasis on the presence of evidence of liver cell failure (for example, ascites, jaundice, lower-limb edema, bleeding tendency, or signs of encephalopathy, in addition to the WHO performance score. Laboratory investigations were done for all patients including liver biochemical profile [s-bilirubin, s-albumin, prothrombin time and concentration, INR, alanine transaminase (ALT) and aspartate transaminase (AST)], blood urea, serum creatinine levels, blood sugar (fasting and 2 hours after food), complete blood count (hemoglobin, white blood cell count, platelet count), coagulation profile (prothrombin time, INR, APPT), α-fetoprotein and hepatitis serologic profile (HBsAg, HBcAb, HCV-Ab, and HCV-RNA by quantitative polymerase chain reaction and HIV). Abdominal ultrasound scanning was done for both groups by using a Hitachi 515 real-time scan after overnight fasting (before and after MSCs transplantation), and MELD score was calculated to assess the degree of hepatic decompensation for every patient.

### Ethics

The ethical committees of Kaser El-Aini School of Medicine and the National Cancer Institute, Cairo University, approved the study protocol, which was in accordance with the Declaration of Helsinki, and a written informed consent was obtained from each patient before enrollment in the study.

### Treatment protocols

**Figure 1:**
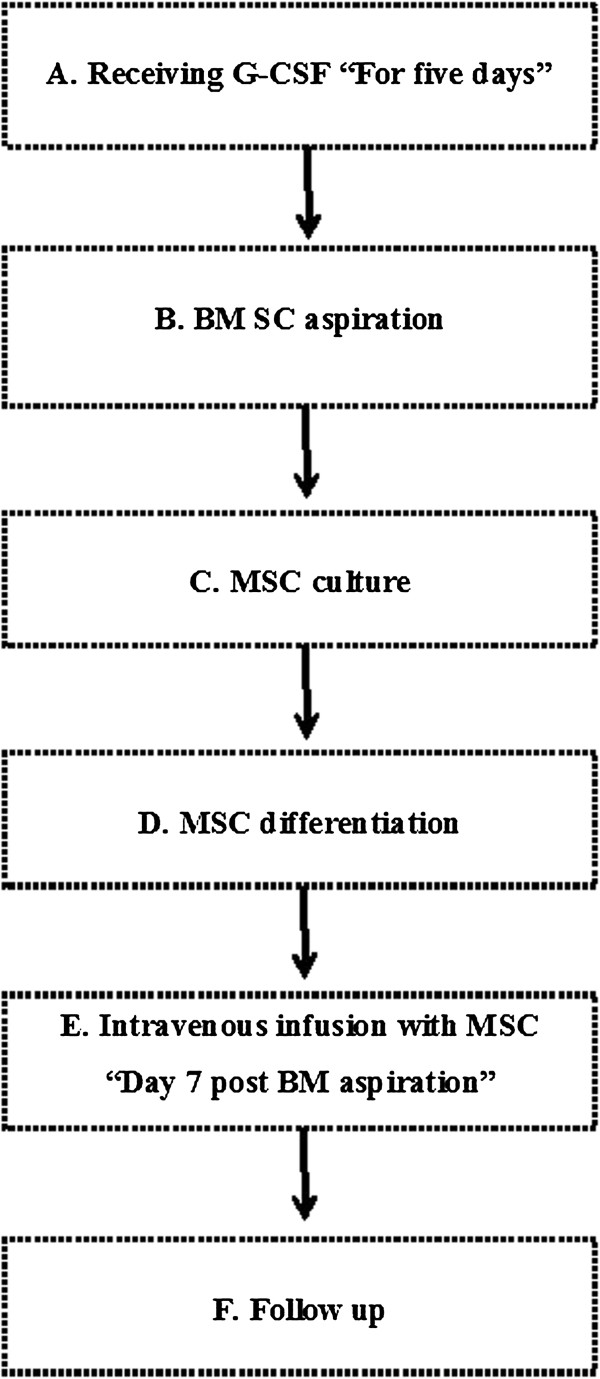
Work flow chart.

A. **Treatment protocol for GI (MSCs transplantation):** Patients in GI received IV MSCs infusion according to the following protocol:

• *G-CSF injection:* All patients received a daily subcutaneous injection of 300 μg of G-CSF (Neupogen; Roche Pharmaceutical) for 5 days to increase the number of the BM-derived stem cells (BMSCs) and hematopoietic stem cells, days 1 to 5.

• *BM stem cell aspiration:* Patient’s skin was cleaned with 70% alcohol at the iliac crest, which is the usual site for puncture in adults. The skin, subcutaneous tissues, and periosteum overlying the selected site for puncture were infiltrated with xylocaine local anesthesia, and serial punctures from multiple sites were performed. With a boring movement, needles (Salah and Klima) were passed perpendicularly into the cavity of the ileum at a point just posterior to the anterior superior iliac spine or 2 cm posterior and 2 cm inferior to the anterior superior iliac spine to aspirate 300 ml of the BM. The collected BM products were transferred to the stem cell laboratory for immunomagnetic purification of CD34+ and CD133+ stem cell population by using the positive cell-selection kit (MACS System Miltenyi Biotec, GmbH, Germany) [11]. The isolated CD34+ and CD133+ cells were washed with phosphate-buffered saline (pH 7.4) supplemented with 0.5% bovine serum albumin and 5 m*M* EDTA, and centrifuged at 1,500 r/min for 10 minutes at 4°C. Cells were counted, adjusted to 0.5 × 10^8^, centrifuged, and resuspended in 100 ml physiological saline.

• MSC differentiation: The cells were cultured in DMEM/HamF12/mesenchymal stem cell media (25%/25%/50%) containing 10% bovine serum albumin (BSA; Invitrogen), 1% penicillin/streptomycin (Invitrogen), 1 ng/ml G-CSF, hepatocyte growth factor (HGF; 5 ng/ml Sigma, H1404), and liver extract (10 ng/ml, Sigma; G7387). Cells were incubated for 5 to 7 days at 37°C in 5% CO_2_ and examined with phase-contrast microscopy for morphologic changes characteristic of MSCs differentiation. A critical point for successful differentiation into MSCs, in the current study, was the relation between the number of cultured cells, the surface area of the tissue-culture flasks used, and the amount of the tissue-culture media.

• Characterization of cultured cells: Cells were periodically examined with phase-contrast microscopy for morphologic changes indicating transdifferentiation into MSCs. Aliquots of cultured cells were also obtained for immunophenotypy characterized with flow cytometry by using surface MSCs markers (CD44-FITC, CD90-PerCP, CD29-FITC, and CD105y versus CD45-PE). Cells were considered MSCs if they were negative for CD45-PE and positive for CD44-FITC, CD90-PerCP, CD29-FITC, and CD105y (either one or all of them)

• Injection of transdifferentiated MSCs: On day 7 after BM aspiration, patients were admitted to hospital while fasting for intravenous infusion with approximately 1 × 10^6^/kg body weight of the expanded MSCs. Figure 1 illustrates the working steps as a simple work-flow chart.

B. **Treatment protocol for GII (regular liver treatment):** Patients in GII received their usual, regular supportive liver treatment

### Follow-up of patients after MSCs infusion

All patients in both groups were followed up every hour for 24 hours, then weekly for the first month, and monthly for 6 months. During the follow-up, the patients were observed for clinical improvement through assessment of the degree of fluid retention (ascites and LL edema), performance status and score, biochemical assessment including s-bilirubin and albumin, prothrombin time and concentration, INR, ALT and AST, blood urea, and s- creatinine levels, blood sugar (fasting and 2 hours after a meal), complete blood analysis, together with assessment of Child-Pugh score progression. Procollagen **III** was evaluated by using human procollagen C-terminal propeptide ELISA (ng/ml) and human procollagen N-terminal propeptide ELISA.

### Statistical analysis

All patients’ data were tabulated and processed with SPSS (Statistical Package for Science and Society) version 12.0 for Windows XP. The descriptive statistics were presented as mean ± SD for quantitative variables, whereas qualitative data were expressed as frequency (number) and percentage. Comparisons between groups were done by using the *χ*2 test, Fischer Exact test, or McNemar test when appropriate for qualitative data. Independent-sample *t* test and paired-sample *t* test were used for normally distributed quantitative variables. The nonparametric Mann-Whitney test and Wilcoxon signed ranks test were used for abnormally distributed quantitative variables. Percentage changes in prognostic variables were calculated and compared by using the Mann-Whitney test. *P* values lower than 0.05 were considered statistically significant. Linear regression analysis was used to determine the degree of improvement of the clinical features in relation to response to treatment**.**

## Results

The baseline laboratory and clinical features of patients in both studied groups are illustrated in Table 1. As for MSCs-injected patients (GI), the baseline mean of s-albumin started to increase 2 weeks after SC infusion (*P* = 0.001), with consistent increase after 1 month (*P* = 0.000), 3 months (*P* = 0.000), and 6 months (*P* = 0.000). All liver-function tests, adjusted for age and sex, improved significantly including bilirubin (*P* = 0.002), ALT (*P* = 0.029), AST (*P* = 0.156) INR (*P* = 0.017), and PC (*P* = 0.029).

**Table 1:** The results of baseline laboratory and clinical investigations of the studied groups

	**Treated group (20)**	**Control group (20)**
	**Mean ± SD**	**Mean ± SD**
Age (years, mean ± SD)	50.27 ± 6.05	50.9 ± 7.23
**Sex**		
Male	17 (85%)	16 (80%)
Female	3 (15%)	4 (20%)
**Residence**		
Urban	7 (35%)	8 (40%)
Rural	13 (65%)	12 (60%)
**Clinical features**		
Hemoglobin (g/dl)	12.22 ± 2.24	9.77 ± 2.13
TLC (1,000/cmm)	5.81 ± 3.22	5.73 ± 2.24
Platelets (1,000/cmm)	105.95 ± 47.17	91.75 ± 33.67
Total bilirubin (N, 0.1 to 1 mg/dl)	1.88 ± 1.05	2.51 ± 0.94
Serum albumin (N, 3.4 to 5.2 g/dl)	2.59 ± 0.28	2.62 ± 0.37
PC	55.34% ± 9.06%	52.85 ± 10.16%
INR (N, 1)	1.53 ± 0.19	1.66 ± 0.33
ALT folds	1.35 ± 0.87	0.9 ± 0.52
AST folds	2.29 ± 1.51	1.81 ± 0.84
Serum creatinine (0.7-1.2 mg/dl)	1.1 ± 0.29	1.06 ± 033
Encephalopathy	1 (5%)	2 (10%)
Ascites and lower-limb edema	15 (75%)	15 (75%)
Bleeding tendency	10 (50%)	10 (50%)
Hematemesis	0 (0)	2 (10%)
**Spleen size**		
Average size	5 (25%)	5 (25%)
Mild splenomegaly	11 (55%)	12 (60%)
Moderate splenomegaly	4 (20%)	3 (15%)
Splenectomy	0	0
**Ascites**		
Absent	9 (45%)	5 (25%)
Mild	2 (10%)	2 (10%)
Moderate	7 (35%)	9 (45%)
Massive	2 (10%)	4 (20%)

None of these parameters showed significant differences between the two groups. ALT, alanine aminotransferase; AST,aspartate aminotransferase; INR, International Normalized Ratio; PC, prothrombin concentration; TLC, total leukocyte count.

**Figure 2:**
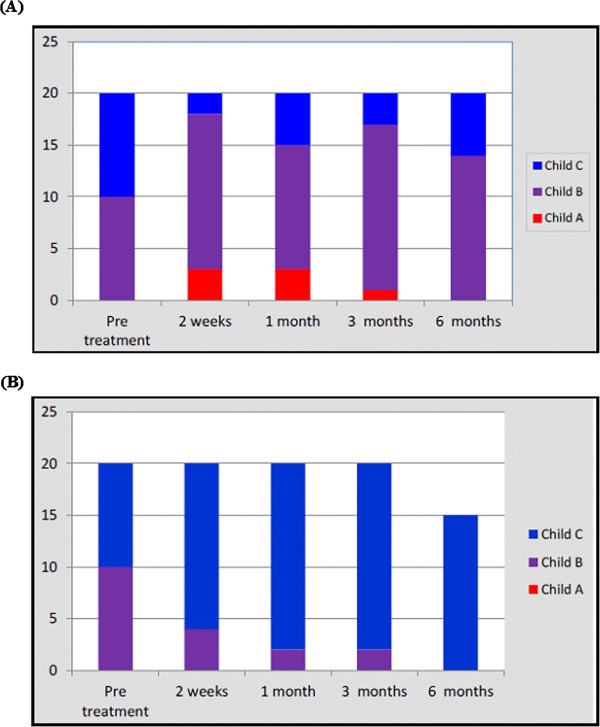
Child-Pugh scores in the study population (A) and control group (B).

No statistically significant difference was found between the studied groups regarding the renal functions at baseline and all through the study. During 6 months of follow-up of the MSCs-treated patients (with ultrasonography, Doppler, and s-AFP), no focal lesions or portal vein thrombosis was detected. No mortality was recorded within 6 months of treatment, although five patients died in the control group during this period. Table 2 illustrates the biochemical changes in both studied groups during the follow-up period.At the end of the study, four patients (20%) showed improvement of their Child-Pugh grade compared with the baseline (Figure 2).

**Table 2:** Changes in the studied groups (mean ± SD)

	**Pretreatment**	**2 weeks**	**First month**	**Third month**	**Sixth month**
**Bilirubin**					
**Treated**	1.88 ± 1.05	1.92 ± 1.22	1.89 ± 1.36	1.82 ± 1.3	2.06 ± 1.26
**Control**	2.51 ± 0.94	2.87 ± 1.5	3.3 ± 2.14	4.02 ± 3.29	4.24 ± 2.48
***P *** **value**	0.4	**0.025**	**0.006**	**0.001**	**0.002**
**Albumin**					
**Treated**	2.59 ± 0.28	3.03 ± 0.44	3.05 ± 0.41	2.99 ± 0.26	3.06 ± 0.36
**Control**	2.62 ± 0.37	2.63 ± 0.27	2.63 ± 0.14	2.63 ± 0.3	2.43 ± 0.36
***P *** **value**	0.77	**0.001**	**0.000**	**0.000**	**0.000**
**PC (%)**					
**Treated**	55.34 ± 9.06	61.15 ± 15.99	62.89 ± 18.2	59.45 ± 15.23	57.59 ± 14.68
**Control**	52.85 ± 10.16	53.65 ± 11.01	49.35 ± 10.35	50.45 ± 11.42	45.03 ± 10.92
***P *** **value**	0.41	0.92	**0.007**	**0.041**	**0.029**
**INR**					
**Treated**	1.53 ± 0.19	1.47 ± 0.29	1.44 ± 0.28	1.47 ± 0.23	1.52 ± 0.36
**Control**	1.66 ± 0.33	1.62 ± 0.39	1.76 ± 0.4	1.73 ± 0.4	1.84 ± 0.39
***P *** **value**	0.12	0.2	**0.007**	0.31	**0.017**
**ASTfold**					
**Treated**	2.29 ± 1.51	2.03 ± 0.79	1.83 ± 1.13	1.97 ± 1.09	2.14 ± 0.88
**Control**	1.81 ± 0.84	1.68 ± 0.65	1.75 ± 0.71	1.66 ± 0.73	1.64 ± 0.69
***P *** **value**	0.46	0.14	0.85	0.48	0.156
**ALT fold**					
**Treated**	1.35 ± 0.87	1.17 ± 0.71	1.1 ± 0.58	1.06 ± 0.64	1.27 ± 0.3
**Control**	0.9 ± 0.52	0.87 ± 0.41	0.81 ± 0.46	0.81 ± 0.54	1.09 ± 1.47
***P *** **value**	0.55	0.13	0.172	**0.093**	**0.029**

ALT, alanine aminotransferase; AST, aspartate aminotransferase; INR, International Normalized Ratio; PC, prothrombin concentration; TLC, total leukocyte count.

### Ascites

In this study, ascites was assessed by abdominal ultrasound examination; ascites only in the pelvis and/or hepato-renal angle is considered mild; If it reaches the mid-abdomen, it is moderate, and more than that is massive ascites. At the beginning of the study, 10% of the MSCs-treated patients (GI) had massive ascites, 35% had moderate ascites, 10% had mild ascites, and 45% had no ascites compared with 20% with massive ascites, 45% with moderate ascites, 10% with mild ascites, and 25% with no ascites in the control group (GII). Two weeks after MSCs infusion, 60% of patients had no ascites, 15% had mild ascites, 20% had moderate ascites, and 5% had massive ascites.

One month later, 65% of the patients had no ascites, 10% had mild ascites, 15% had moderate ascites, and 10% had massive ascites compared with 60% with no ascites, 5% with mild ascites, 15% with moderate ascites, and 20% with massive ascites after 3 months.

After 6 months of follow-up, 65% of the patients had no ascites, 5% had mild ascites, 10% had moderate ascites, and 20% had massive ascites.

In the control group, 25% of the patients had no ascites, 10% had mild ascites, 45% had moderate ascites, and 20% had massive ascites after 2 weeks compared with 15% with no ascites, 15% with mild ascites, 55% with moderate ascites, and 15% with massive ascites after 1 month of follow-up. Three months later, 10% of the patients had no ascites, 15% had mild ascites, 30% had moderate ascites, and 45% had massive ascites. After 6 months, 13.3% of the patients had mild ascites, 40% had moderate ascites, and 46.7% had massive ascites (*P* = 0.07; *P* = 0.001).

### Hepatic encephalopathy

At the beginning, 95% of the patients in the MSCs-treated group were free of encephalopathy attacks compared with 75% in the control group (*P* = 0.18). After 2 weeks, 90% of the MSCs-treated patients were free of attacks of encephalopathy compared with 75% in the control group (*P* = 0.4), and after one month, 95% of the patients in MSCs treated group had no attacks compared to 70% in the control group (*P* = 0.09). After 3 months of follow-up, 85% of the patients in MSCs-treated group had no attacks of encephalopathy compared with 60% in the control group (*P* = 0.77), and after 6 months, 75% of patients in the MSCs-treated group had no attacks compared with 46.7% in the control group (*P* = 0.08). No statistically significant difference was noted between both studied groups during the whole follow-up period.

### Hematemesis

At the beginning of the study, 100% of patients had no history of hematemesis in the MSCs-treated group compared with 90% in the control group (*P* = 0.48) compared with 100% and 95%, respectively, in both studied groups after 2 weeks and 1 month of follow-up (*P* = 1.0). After treatment, no further attacks of hematemesis occurred in the MSCs-treated group 3 or 6 months after MSCs infusion, whereas 15% and 26.7% of the patients in the control group had attacks of hematemesis at 3 and 6 months, respectively, because of bleeding esophageal varices. The difference between the two groups was statistically significant after 6 months (*P* =0.026) but not after 3 months (*P* = 0.23).

Regarding the mortality rate, no mortality was reported in the MSCs treated group while five patients in the control group died (all were child C), three of them due to hepatic encephalopathy and two due to un-controlled attacks of haematemesis.

### Fibrosis biomarkers

**Figure 3:**
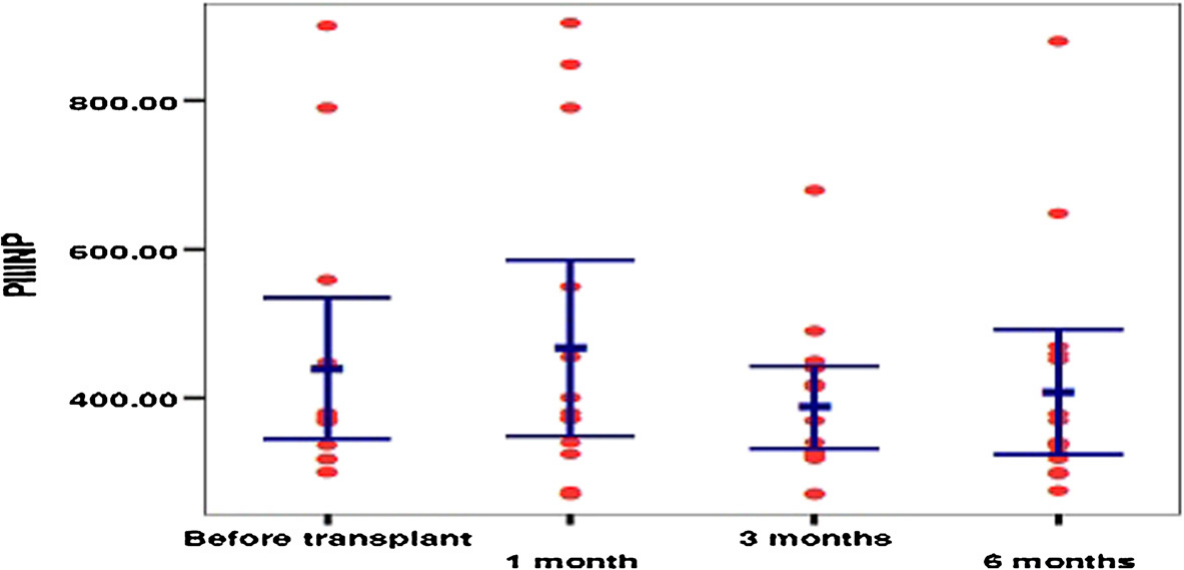
Scattered plot distribution of PIIINP concentration in treated group before transplant, after 1 month, after 3 months, and after 6 months.

**Figure 4:**
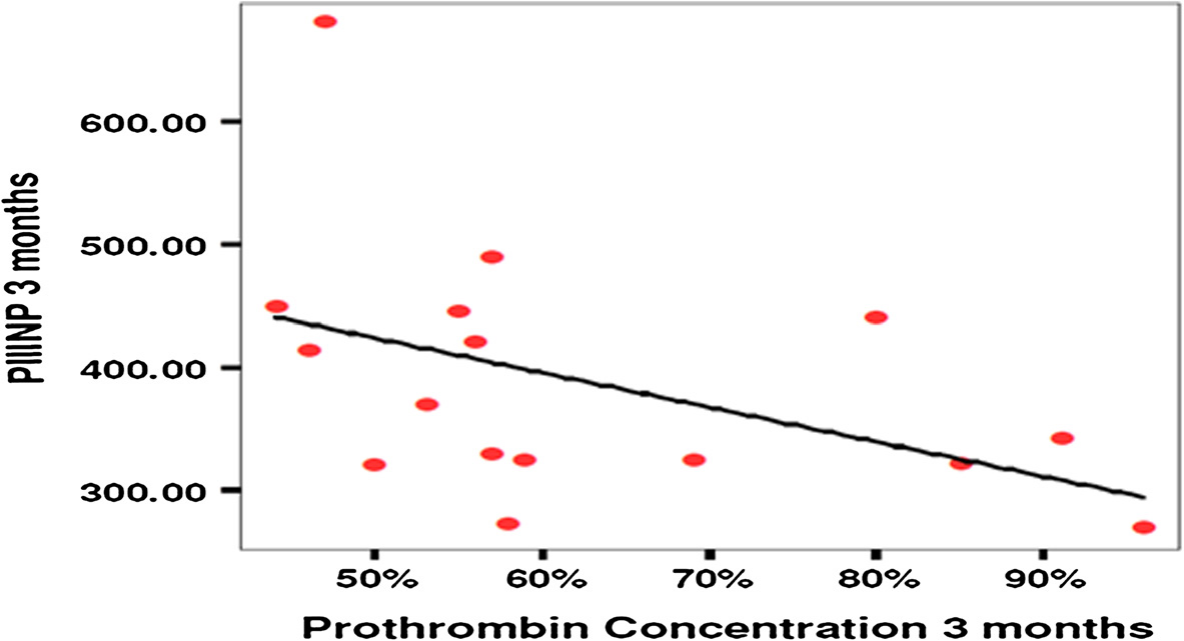
Correlation coefficient between PIIINP and prothrombin concentration.

The mean pretreatment serum baseline level of the PIIINP was 440 ± 189, then 468 ± 212, 388 ± 102, then 408 ± 156 after 1 month of treatment, 3 months, and at the end of the study, respectively. The difference was statistically insignificant (*P* = 0.4) (Figure 3). Conversely, the difference in s-PIIINP level and PC (prothrombin concentration) before and 3 months after treatment with IV-MSCs infusion was statistically significant (*P* < 0.05) (Table 3, Figure 4).

**Table 3:** Correlation coefficient between PIIINP and the following parameters

	**Pretreatment**	**1 month**	**3 months**	**6 months**
Albumin	0.06	-0.04	0.3	0.2
INR^(a)^	-0.5	-0.1	0.4	0.4
PC^(b)^	0.5	0.1	-0.5*	-0.3
Ascites	0.03	0.2	0.2	0.04

*There is significant correlation coefficient between PIIINP and Prothrombin concentration after 3 months.

^a^: INR, International Normalized Ratio; ^b^: PC, prothrombin concentration.

**Figure 5:**
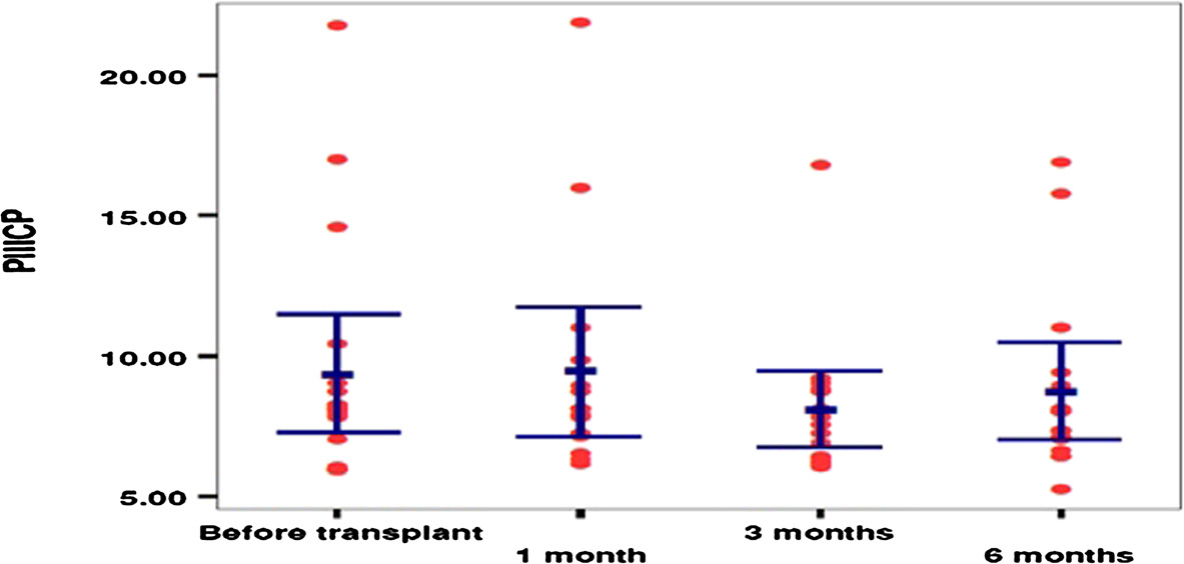
Scattered plot distribution of PIIICP concentration in the treated group before transplant, 1 month, 3 months and 6 months.

**Figure 6:**
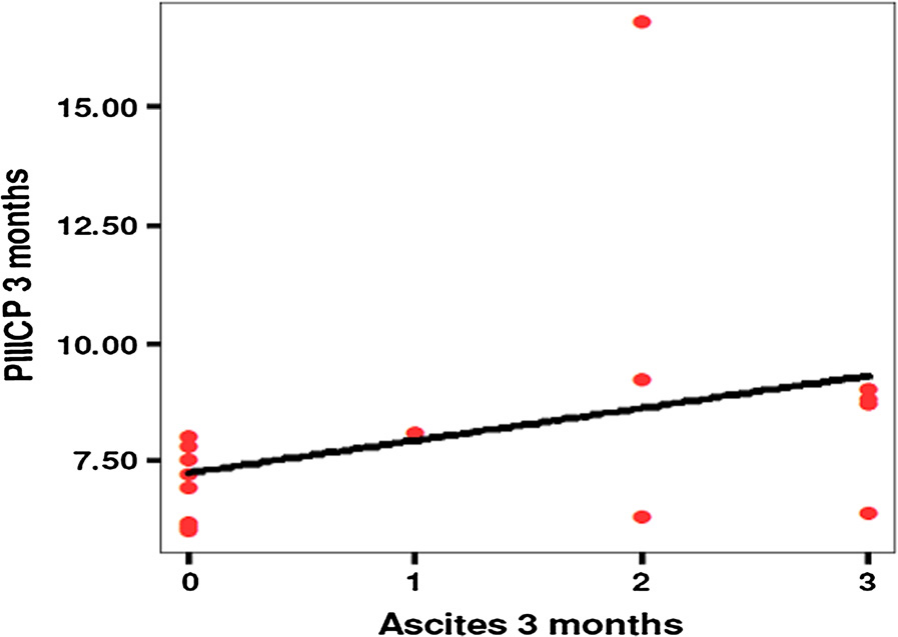
Correlation coefficient between PIIICP and ascites.

**Figure 7:**
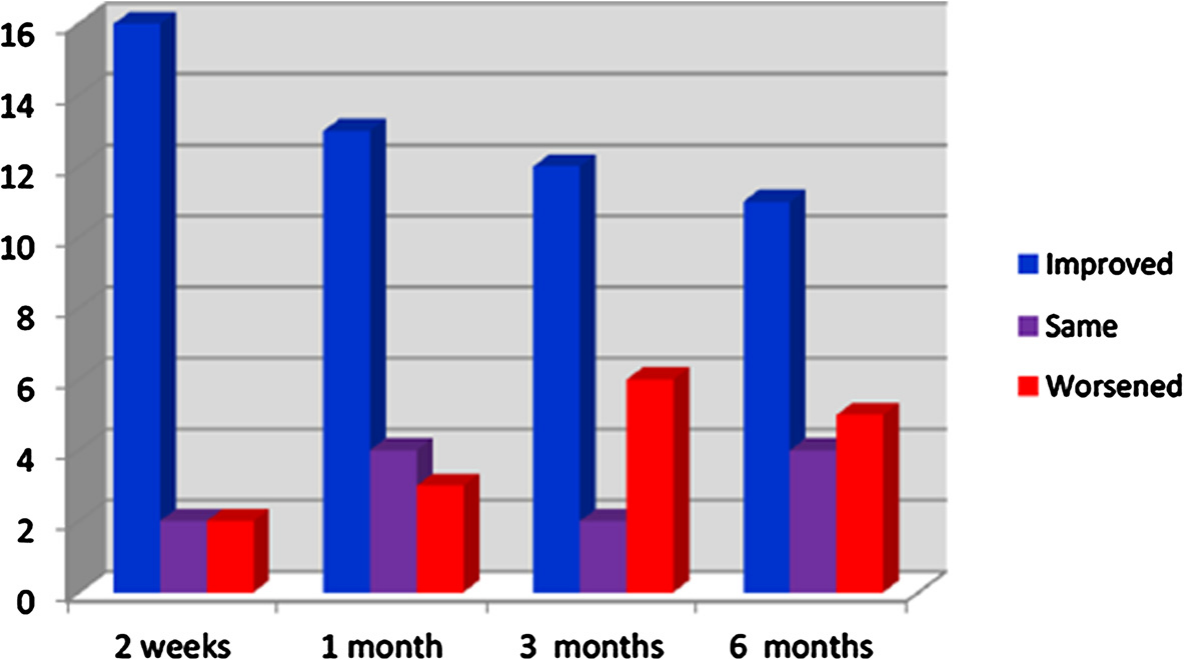
Performance-score difference after 2 weeks, 1, 3, and 6 months in treated group.

The mean pretreatment serum baseline level of the PIIICP was 9.4 ± 4.25; then it was 9.4 ± 4.2 after 1 month of treatment, 8.1 ± 2.6 after 3 months, and 8.7 ± 3.1 at the end of the study. The difference was statistically insignificant (*P* = 0.7) (Figure 5). However, A significant correlation was reported between s-PIIICP and ascites after 3 months of treatment with IV-MSCs (*P* = 0.550*) (Table 4, Figure 6).Health quality-of-life assessment showed a statistically significant improvement in the MSCs-treated patients started in the second week after treatment until the end of the study (Figure 7). Similarly, improvements in hepatic coma and hematemesis were detected in all treated patients compared with the control group.

**Table 4:** Correlation coefficient between PIIICP and the following parameters

	**Pretreatment**	**1 month**	**3 months**	**6 months**
Albumin	0.3	-0.07	0.08	0.3
INR^(a)^	-0.2	-0.1	0.14	0.3
PC^(b)^	0.15	0.16	-0.3	-0.3
Ascites	0.12	0.3	0.550*	0.1

*There is significant correlation coefficient between PIIICP and ascites after 3 months.

^a^: INR, International Normalized Ratio; ^b^: PC, prothrombin concentration.

## Discussion

Cirrhosis represents the final pathologic outcome for the majority of chronic liver disease cases. Until now, liver transplantation has been considered the only curative treatment for decompensated cirrhosis [12]. However, this procedure is limited by technical difficulties, high cost, and also by a lack of donors [13]. In many recent studies, some done by our team, have shown that BMSCs transplantation can restore liver mass and function, alleviate fibrosis, and correct inherited liver diseases [6,14]. In the current study, we evaluated the efficacy of peripheral vein infusion MSCs followed G-CSF mobilization as a treatment for patients with end-stage liver disease, and we confirmed our previous data regarding the supportive role of SC transplantation in patients with end-stage liver disease.

The SC therapy is affected by many variables, including the type and number of transplanted cells, the culture media used, and the growth factors which are added to the culture media to support growth and differentiation of cells, the administration route, and the posttreatment care. In the current study, the treated group received 300 μg G-CSF daily for 5 days. The G-CSF is effective in mobilizing bone marrow cells into the peripheral blood and then homing into the liver [12]. Studies in rats demonstrate that G-CSF induces liver repair by increasing the BM-derived liver repopulation, and activating the endogenous oval cells, which express G-CSF receptor (G-CSFR) [15]. The G-CSF mobilized peripheral bone marrow cells (PBMCs) secrete chemokines might enhance the transplanted PBMCs, which are located in the injured liver and cytokines that enhance the proliferation of the transplanted cells or hepatic stem cells, and they also induce apoptosis of liver stellate cells [15,16]. It is now clear that the modulation of hepatic stellate cells’ activity is one of the main causes of liver cirrhosis, and this is through collagen deposition, vasoconstriction, and regulation of sinusoidal structure, which are the results of the changes in stellate cells’ activation**.** Consequently, reversing these changes in hepatic stellate cells activity**,** induction of apoptosis**,** inhibition of matrix formation, as well as degradation of established matrix could be the strategy for inhibition of liver cirrhosis progression [17-20].

In this study, we used the MSCs, as we think that they represent an excellent extrahepatic source for hepatocytes to induce liver regeneration. As previously shown, the MSCs play an important role in the molecular signaling during early development of the liver [21,22]. They express a number of growth-factor receptors, such as fibroblast growth factor receptor (FGFRs), and the receptor for HGF (c-met) [23,24], and they also induce alterations in the dendritic cells (DCs) cytokines secretion [25,26]. As a result, they upregulate the antiinflammatory Th2 cytokines (IL-3, 5, 10, and 14) and downregulate the proinflammatory Th1 cytokines (IL-1α and, IFNγ, and TNFα) [26,27].

It was also shown that the MSCs may act directly as an anti-collagen formation agent rather than an immune regulator. MSCs can correct the liver functions through downregulation of the collagen matrix formation. The results of the current study confirm previously published data regarding MSCs functions, since we reported a decrease in serum levels of the hepatic fibrosis markers, PIIICP and PIIINP, in the MSCs-infused patients. Similar findings were also reported by Terai *et al*. [5]. Taken together, this indicates that downregulation of collagen matrix formation reflects an increase in serum albumin level and reduces ascites. All of these factors make us think of the MSCs as the perfect choice for liver stem cell therapy, as they could help in different developmental stages of the liver and restoring liver functions.

In the current study, five patients (25%) showed an improvement of the grade of ascites, as assessed by abdominal ultrasound, compared with the baseline. This is in agreement with the results of Terai *et al*. [5], Gordon *et al*. [28], and Pai *et al*. [14] by using autologous mesenchymal bone marrow transplantation and Salama *et al*. [9] by using autologous CD34+ and CD133+ stem cell infusion.

Our data regarding the improvements in liver functions and ascites also support the utility of MSCs. We found that, in more than 75% of the MSCs-infused patients s-albumin started to improve 2 weeks after the procedure and until the end of the study without albumin infusion compared with the control group. The difference between the two groups was statistically significant difference, which is in agreement with our previous studies [9,10] as well as with those of Gordon *et al*. [28] and Terai *et al*. [5] using autologous bone marrow cell infusion (ABMI). In addition, we have observed improvement in the ALT, AST, and S-albumin levels, which is in agreement with our previous data using autologous CD34+ and CD133+ stem cell infusion [9], as well as Gordon *et al*. [28] and Lyra *et al*. [29].

As for the hepatic functional reserve**,** our data showed improvement in the Child-Pugh score as well as in the performance score (PS), which led to improvement in the quality of life in the MSCs-treated group, which started 2 weeks after the procedure until the end of the study, which is in consistent with those of with our previous study using autologous CD34+ and CD133+ stem cell infusion [9] as well as Terai *et al*. [5], Pai *et al*. [14], and Gordon *et al*. [28].

No agreement exists regarding the cell number of therapeutic stem cells to be infused. In previous studies, authors had used different doses: 1 × 10^5^ embryonic stem cells [30] and 31.73 × 10^6^ autologous BMSCs for LC patients [31]. In an earlier study, we found that a high dose (1 × 10^11^) was not suitable for the patients [10], as it could lead to portal hypertension and lung edema; therefore, in the current study, we injected all patients with 1 × 10^7^ cells/kg of body weight. Although this dose is higher than any previously injected doses, it was tolerable and proved to be very safe and without any clinical complication on follow-up.

Similarly, one of the aims of this study was to assess the efficiency of the peripheral-vein infusion as a route of administration of the MSCs after G-CSF mobilization. It also proved to be safe and superior to other previously used routes. The peripheral intravenous infusion is an easy and convenient way of stem cells delivery, with less-invasive and less-traumatic effects, and it does not need advanced medical equipment. However, as a systemic administration, concerns exist of the possible side effects, such as fever and immunoreaction. Our study provided that the peripheral vein infusion is as evidenced by the low incidence of complications encountered after 6 months’ follow-up of the treated group by ultrasonography, Doppler, and serum α-fetoprotein. No focal lesions or portal vein thrombosis were detected after the procedure. This is in agreement with Mohamadnejad M *et al.*[30], who previously showed that MSCs can differentiate into hepatocyte and nonhepatocyte cells and that MSCs infusion through a peripheral vein can prevent liver cell failure and lead to regression of liver fibrosis. When we compared the peripheral vein infusion to the intrahepatic infusion, which we used in our previous studies [9,10]; we did find any significant differences either in the overall clinical outcome or in the incidence of complications, between the two used procedures. We have also observed in this study that the serum albumin has been improved faster than that in our previous studies [9,10].

## Conclusion

**Figure 8:**
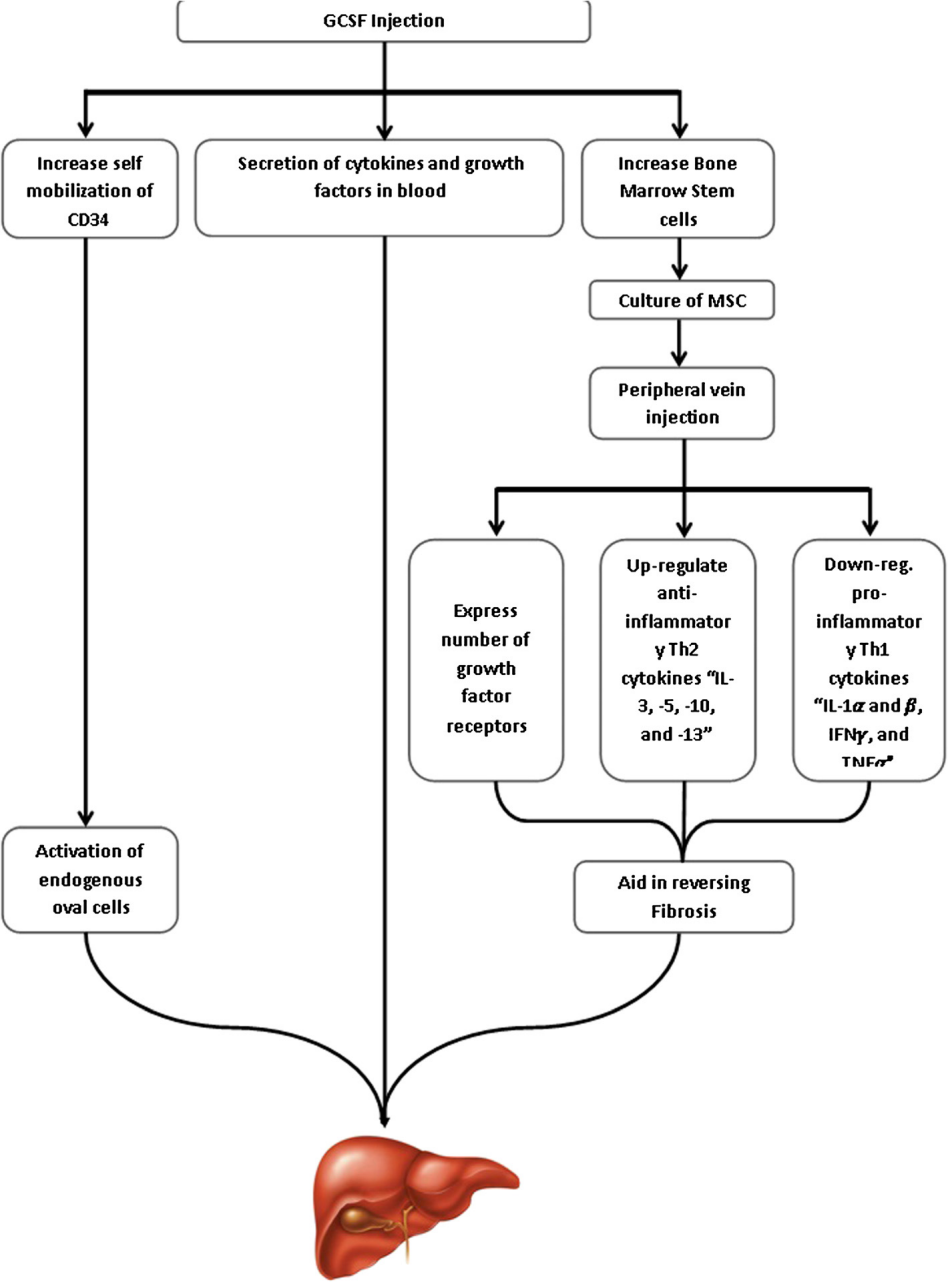
This diagram illustrates our assumption of concomitant mode of action of both GCSF priming followed by MSC transplantation for regeneration of liver cells in end-stage liver disease.

We conclude that (a) a combination of G-CSF with MSCs will greatly improve the outcome of stem cell-treated patients with end-stage liver disease. (b) The intravenous route is a safe and easy way for MSCs infusion in end-stage liver disease and with fewer complications compared with intraarterial infusion route, and (c) MSCs may act directly through preventing collagen formation, as evidenced by their ability to reduce the hepatic fibrosis markers. Taken together, our data provides evidence that MSCs followed GCSF mobilization is excellent for liver stem cell therapy to retain liver mass and restore liver functions (Figure 8).

## Abbreviations

ABMI: Autologous bone marrow cell infusion; AFP: α-fetoprotein; ALT: alanine transaminase; AST: aspartate transaminase; BM: bone marrow; BMSCs: bone marrow stem cells; c-Met: HGF receptor; DC: dendritic cell; ELISA: enzyme-linked immunosorbent assay; FGF: fibroblast growth factor; FGFR: fibroblast growth factor receptor; G-CSF: granulocyte colony-stimulating factor; G-CSFR: granulocyte colony-stimulating factor-receptor; HBcAb: hepatitis B core antibody; HBsAg: hepatitis B surface antigen; HCV Ab: hepatitis C antibody; HCV: hepatitis C virus; HGF: hepatocyte growth factor; HSCs: hematopoietic stem cells; INR: increased international normalized ratio; MSCs: mesenchymal stem cells; PBMCs: peripheral bone marrow cells; PC: prothrombin concentration; PIIICP: procollagen III C terminal peptide; PIIINP: procollagen III N-terminal peptide; PS: performance score; PT: prothrombin time; SC: stem cell; SD: standard deviation; SPSS: Statistical Package for Science and Society; WHO: World Health Organization.

## Competing interests

The authors declare that they have no competing interests.

## Authors’ contributions

HMS shared in the study design and managed the stem cell-treated patients. ARNZ generated the idea and was responsible for the stem cell separation and differentiation, data analysis, and editing and revising the manuscript. EM shared in the study design and was also the responsible clinician for treating the control group and helped in the clinical management of the stem cell-treated patients as well. SAA was involved in stem cell patient infusion and the acquisition of data from stem cell-treated patients and the control group. OSA helped in the stem cell isolation and differentiation in the stem cell laboratory and followed up the culture. AAB was responsible for flow cytometry laboratory for stem cell characterization, stem cell isolation, and differentiation, RT-PCR of differentiated cells and helped in editing the manuscript. MML helped in editing and revising the manuscript for intellectual content and figure layout. RA helped in the management of chronic hepatitis patients and the control group. SM shared in the management of the chronic active hepatitis patients and the control group. All authors were involved in drafting the work and revising it critically for important intellectual content. All authors agree to be accountable for all aspects of the work in ensuring that questions related to the accuracy or integrity of any part of the work are appropriately investigated and resolved. All authors read and approved the final manuscript.
